# Microfluidic cell isolation technology for drug testing of single tumor cells and their clusters

**DOI:** 10.1038/srep41707

**Published:** 2017-02-02

**Authors:** Swastika S. Bithi, Siva A. Vanapalli

**Affiliations:** 1 Department of Chemical Engineering, Texas Tech University, Lubbock, TX 79409, United States.

## Abstract

Drug assays with patient-derived cells such as circulating tumor cells requires manipulating small sample volumes without loss of rare disease-causing cells. Here, we report an effective technology for isolating and analyzing individual tumor cells and their clusters from minute sample volumes using an optimized microfluidic device integrated with pipettes. The method involves using hand pipetting to create an array of cell-laden nanoliter-sized droplets immobilized in a microfluidic device without loss of tumor cells during the pipetting process. Using this technology, we demonstrate single-cell analysis of tumor cell response to the chemotherapy drug doxorubicin. We find that even though individual tumor cells display diverse uptake profiles of the drug, the onset of apoptosis is determined by accumulation of a critical intracellular concentration of doxorubicin. Experiments with clusters of tumor cells compartmentalized in microfluidic drops reveal that cells within a cluster have higher viability than their single-cell counterparts when exposed to doxorubicin. This result suggests that circulating tumor cell clusters might be able to better survive chemotherapy drug treatment. Our technology is a promising tool for understanding tumor cell-drug interactions in patient-derived samples including rare cells.

Understanding interactions between tumor cells and drugs is important for discovery of new oncogenic targets^1^,^2^,^3^, development of cancer drug candidates^4^ and generating insights into the mechanisms of chemotherapy drug resistance^5^,^6^. Despite significant advances in understanding mechanisms of tumor development and progression^7^,^8^, the current clinical success rate of lead cancer drug candidates remains below 5%, significantly lower than that of cardiovascular (~20%) and infectious diseases (~17%) therapies^2^. Likewise, chemotherapy drug resistance is believed to be responsible for treatment failure in more than 90% patients with metastatic disease^9^, motivating the need to better understand in a patient-specific manner how chemotherapy drugs interact with cancer cells so that personalized treatments can be designed.

Identifying new drug targets or compounds and the molecular mechanisms of chemotherapy resistance requires preclinical models that adequately capture the complexities of cancer. Established tissue culture cell lines are often used as an *in vitro* model of cancer^10^,^11^,^12^, but these cell lines display amplified proliferation, transformed sensitivity to chemotherapy, and reduced cellular heterogeneity^13^,^14^,^15^. As a result, there has been a growing interest in conducting drug studies with patient-derived cells including human tissues and biofluids as a superior model of the *in vivo* situation^10^,^13^,^16^. Patient-derived cells are expected to better predict patient outcomes as they have been found to be more heterogeneous, with reduced proliferation rates and enhanced resistance to chemotherapy compared to established cell culture lines^17^.

Among the patient-derived cells, circulating tumor cells (CTCs) isolated from the blood of cancer patients offer a rich test bed for drug development and chemoresistance assays because (i) CTCs and their clusters (of typically 2–50 cells^18^,^19^,^20^) provide a compelling mechanism for metastasis^19^, with clusters having significantly more metastatic potential^19^, (ii) molecular profiling of CTCs shows they are very heterogeneous, similar to cells in a primary tumor, and share some common genetic mutations^21^,^22^, (iii) blood samples are less invasive compared to tissue biopsies and are easier to procure, and (iv) they can be sampled longitudinally for identifying drug resistance. Thus, CTCs are an attractive candidate for drug discovery and probing mechanisms of chemoresistance.

The promise of CTCs for drug investigations has been complemented by an explosion in the number of available microfluidic technologies available for isolating CTCs, even though they are present in low counts, typically 1–100 cells per mL of blood^23^. A number of microfluidic techniques are capable of antibody-based capture and release of CTCs^24^,^25^,^26^. In addition to these immunocapture methods, several label-free methods based on size and deformability also exist to separate CTCs^25^,^27^,^28^,^29^. More recently, clusters of CTCs have also been isolated using microfluidic approaches^30^.

The advent of numerous technologies for efficiently isolating CTCs opens unique opportunities for using CTCs for drug discovery and probing drug resistance. However, technical hurdles still exist for conducting drug investigations using CTCs. First, even though microfluidic technologies are available for efficiently isolating and collecting CTCs, conducting drug assays downstream can be challenging due to potential loss of the rare cells while handling them using pipettes and multiwell plates. Second, although *ex vivo* culture methods are beginning to emerge to culture CTCs for drug assays^31^,^32^,^33^, the molecular heterogeneity of individual CTCs and clusters is often lost during the bulk expansion process making it difficult to identify drug resistant cells.

In this study, we present a pipette-based *microfluidic cell isolation* (MCI) technology that is capable of conducting single cell resolution drug assays with a small number of tumor cells or their clusters present in small sample volumes (e.g. 10–100 cells in 10 μL). The method is based on digitizing the sample volume containing tumor cells into an array of nanoliter-scale droplets by simply using a pipette and a microfluidic device. The sample digitization occurs in the device in such a way that an array of static droplets is created in which tumor cells and their clusters are isolated. This approach also allows automated imaging of tumor cells stored in the droplets.

To establish proof-of-principle of our pipette-based MCI method for CTC research, we use breast cancer cells (MCF-7) and a chemotherapy drug, doxorubicin. Doxorubicin is an FDA approved cytotoxic drug used widely in cancer chemotherapy^34^,^35^ and this was chosen in this study as it is the most active single agent available for the treatment of breast cancer^36^. Using this system, we demonstrate that (i) individual MCF-7 cells can be isolated without any loss during the pipetting and digitization steps (ii) our method can isolate clustered tumor cells containing 2–22 cells per cluster (iii) the uptake profile of doxorubicin is heterogeneous in individual tumor cells and that a critical amount of intracellular uptake of doxorubicin determines the onset of apoptosis (iv) clustered tumor cells show a higher viability than their single-cell counterparts when exposed to doxorubicin suggesting that being in a cluster can alter a cancer cell’s response to chemotherapy drugs. The overall viability of clusters might be improved due to the coordinated resistivity provided by the group of cells. Overall, our MCI technology is a promising tool for chemotherapy drug testing on CTCs and their clusters.

## Results and Discussion

### Basic description of the MCI technology

In recent years, drop-based microfluidics has emerged as a powerful technology to compartmentalize cells in volumes down to picoliters^37^,^38^,^39^. The standard practice has been to encapsulate cells within trains of individual droplets produced in T-junction or flow-focusing devices, using syringe pumps. Subsequently, these droplets are stored in a variety of ways including on-chip incubation chambers/traps^40^,^41^ and off-chip incubation^39^. Although such methods have been shown to efficiently isolate individual cells for single-cell assays^39^, these techniques are incompatible with the needs of rare cell handling and manipulation, where only 10–100 cells are present in about 10–100 μL of sample volume. Specific reasons for the incompatibility include (i) establishing a stable flow and consistent drop production by syringe pumps requires stabilization time, which necessitates the use of more sample volume, typically >0.1 mL (ii) the inevitable presence of dead volumes resulting in not all of the sample being digitized into droplets and (iii) finally, there is a chance of losing cells in the syringe and tubing due to sedimentation and non-specific adhesion. Thus, the standard practice of using syringe pumps for droplet generation is ill suited for compartmentalizing rare cell samples.

**Figure 1:**
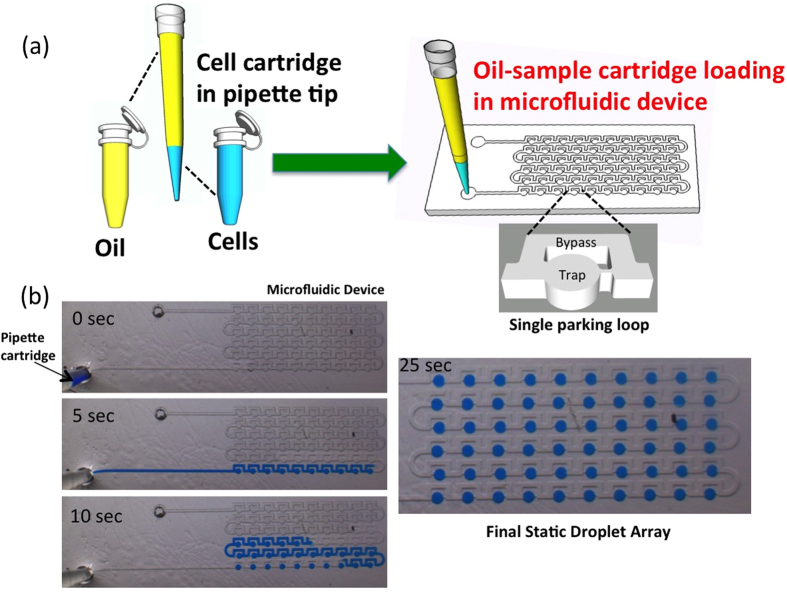
Basic principle of operation of the pipette integrated microfluidic cell isolation technology. (**a**) Schematic showing preparation of cartridge by sequential aspiration of oil and cell-laden sample, followed by a single step dispensing into the microfluidic device that has been prefilled with oil. The microfluidic device contains 60 parking loops. Each loop has a bypass channel and a lower branch containing a trap that can park a droplet of volume 30 nL. (**b**) Time-stamped images of plug motion through the device. The sample plug (blue dye solution) fills the channels and traps and is sequentially digitized by the oil plug producing an array of static droplets of uniform size.

We describe a simple method that resolves the above issues making it particularly suited for assaying rare cells. As shown in Fig. 1, our *MCI* technology involves using a pipette as a fluid-handling tool^42^ in conjunction with a microfluidic device that is capable of storing or parking arrays of nanoliter droplets. This approach is much more simplified than techniques presented in our previous work, where we used syringe pumps to create droplet arrays^43^,^44^. The current technique involves a highly robust method of creating an off-chip oil-sample cartridge in a pipette tip by sequentially aspirating oil and sample, followed by a single step dispensing into the microfluidic device that has been prefilled with oil.

The mechanism of sample digitization is dictated by the geometry of the microfluidic parking network^43^,^44^,^45^. As shown in Fig. 1b, the device contains several interconnected parking loops, with each loop containing a trap chamber and a bypass channel. As the plug travels through the microfluidic device, the long aqueous plug breaks at the junctions of the parking loop, leaving remnants of the plug parked in the trap chambers. The manual pipetting does not introduce large error in droplet volumes since the trapped volume is preset by the size of the trap. This process creates an array of sixty drops of uniform volumes (30 nL in Fig. 1b, with a polydispersity of <5%) in less than 30 sec, using only 2 μL sample. Thus, our *MCI* approach enables the production of static droplet arrays (SDAs) with 90% of the sample being digitized and stored on the device, by simply using a pipette and avoiding the complications of syringe pumps.

### Isolation of single tumor cells (Sl-TC)

**Figure 2:**
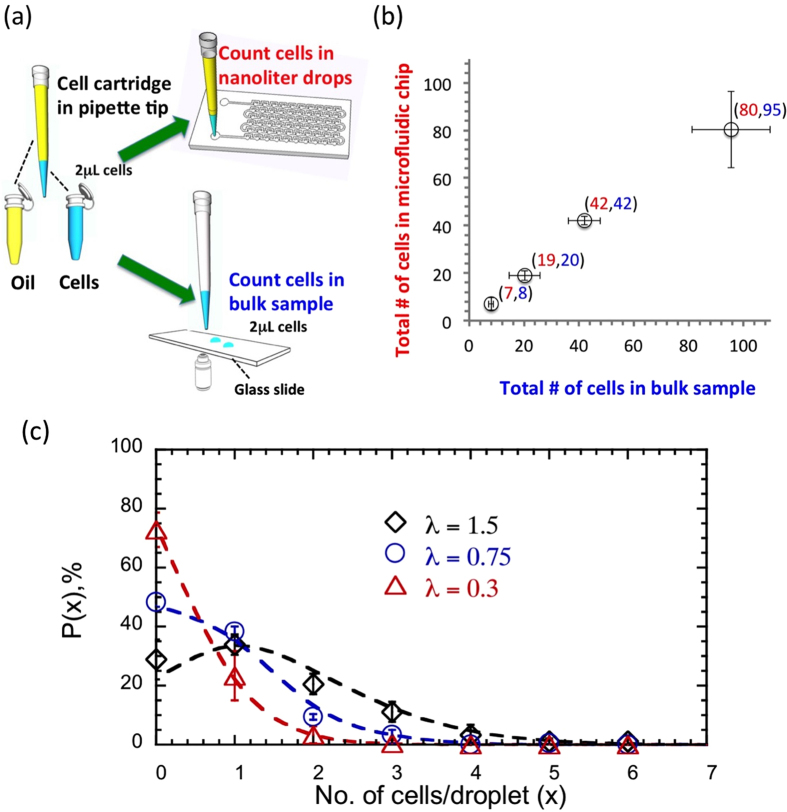
Testing loss-less separation of cells from small sample volumes using the microfluidic cell isolation technology. (**a**) Experimental protocol used to test the loss-less separation of MCF-7 cells. Cell samples were drawn from four serially diluted solutions and their concentration was measured by pipetting 2 μL on a glass slide and counting the number of cells. The same cell samples were loaded into the microfluidic device. The number of cells in each 30 nL drop was counted to determine the total number of cells in the microfluidic device. (**b**) The results from the bulk off-chip sample analysis (n = 5) are compared with the counts obtained from microfluidic cell separation (n = 3). The numbers in brackets denote the actual cell counts obtained from both the methods. The error bars represent standard deviation resulting from the trials conducted. (**c**) Efficacy of isolating single cells using microfluidics. For three different cell samples, the number of cells were counted in each 30 nL drop, and a probability distribution was generated from a total of 60 drops. The error bars represent standard deviation from n = 3 trials. The lines are predicted curves from Poisson distribution.

Given that we can efficiently digitize the sample volume, we tested our approach as to whether (i) cells can be stored in the device without loss during sample manipulation, and (ii) single cells can be efficiently isolated in individual drops. This testing is important to ensure that rare CTCs can be isolated individually without cell loss. As a proof-of-principle, we used the human breast cancer cell line MCF-7. Experimental protocols used to test the efficacy of isolating MCF-7 cells are schematically illustrated in Fig. 2a. Four serially diluted solutions (500, 250, 100, and 50 cells per 10 μl) of cell samples were prepared from a stock solution of 3.5 × 10^6^ cells/mL. These diluted samples were analyzed for cell counts both off-chip and in the device. For off-chip analysis, 2 μL of cell samples were pipetted on a glass slide and the number of cells were counted. For on-chip analysis, cell cartridges were prepared by aspiration of 5 μL of the biocompatible oil (FC-40) followed by aspiration of 2 μL of cell samples. This cell cartridge was then hand-dispensed into the microfluidic device that was preloaded with the biocompatible oil. The number of cells in each 30 nL drop was counted to determine the total number of cells in the microfluidic device. The results from the bulk sample analysis (n = 5) are compared with the counts obtained from microfluidic cell separation (n = 3) (Fig. 2b).

As shown in Fig. 2b, the data obtained from the two methods are in very good agreement. For example, the MCI method has 0% loss for the sample with 250 cells/10 μl, 5% loss for the sample with 100 cells/10 μl and maximum 15% loss for the sample with 500 cells/10 μl. Thus, the MCI technology showed 85–100% effectiveness for loss-less separation of cells from small sample volumes. Our results suggest that there is little or no adhesion of cells to pipette tips. However, a recent study suggests that nonspecific cell adhesion can occur due to the hydrophobic nature of most commercially available pipette tips^46^. We believe that non-specific adhesion of cells to pipette tips is mitigated in the MCI approach due to the presence of a thin biocompatible oil film in the pipette tip, which prevents cell loss during pipette-based fluid handling.

We also tested whether our method for single cell isolation produces Poisson statistics. Previously, single particle encapsulation in microfluidic droplets using T-junction and flow-focusing devices^40^ have shown that the encapsulation process follows a Poison distribution:


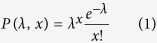


where *x* is the number of cells present in the droplet and λ is the expected average number of cells per droplet, which is adjusted by controlling the stock cell density.

In microfluidic droplet generation methods such as T and cross-junction, cells are encapsulated at sub-second time scales, however, in our case the timescale for encapsulating cells in droplets is tens of seconds (see Fig. 1b) causing potential cell sedimentation issues^44^, which might alter the cell encapsulation statistics from a Poisson distribution. To test the applicability of Poisson statistics for the *MCI* method, we used three different cell samples (500, 250, 100 cells/10 μl). For these cell concentrations, the expected average numbers of cells per droplet (λ) are 1.5, 0.75, and 0.3. The probability distribution P(x) was calculated by counting the number of cells per droplet (*x*) from a total of 60 drops for each cell sample. Using Eqn. (1), we also estimated the theoretical Poisson distribution considering the number of cells/per trap (*x* = 0, 1, 2, 3, 4…..) for each expected average number of cells per droplet (λ). Figure 2c shows the statistics of the isolation process from the experiment (symbols) and it’s comparison to theoretical prediction (dashed lines). We find that the experimental cell distribution closely follows Poisson statistics and about 25–35% drops in the microfluidic device contain single cells.

Overall, the MCI technology is capable of efficient isolation of individual cells from minute samples that contain a small number of cells, without much cell loss. The MCI approach addresses the current issues associated with handling rare cells using pump-based droplet microfluidics. Likewise, it outperforms traditional pipette and multiwell plate fluid handing method that have difficulty in handling nanoliter volumes and isolating single cells. Thus, the MCI technology has significant potential for handling small number of cells in minute samples that is typical for assays with isolated CTCs or primary human cells.

### Influence of microfluidic drop environment on tumor cell viability

**Figure 3:**
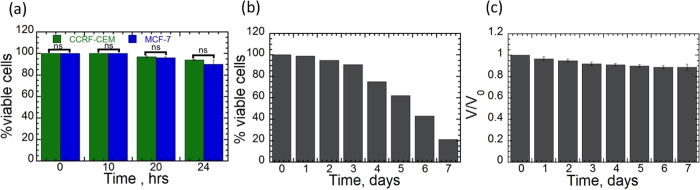
Viability of tumor cells in drop environment. (**a**) Comparison of cell viability of MCF-7 breast tumor cells and CCRF-CEM leukemia cells in the liquid drop environment, measured over 24 hrs. For all time points, the difference in viability between the two cell lines is not statistically significant (p > 0.05: ns (not significant)) suggesting that the level of anoikis is not very different between the two cell lines. The error bars represent standard deviation from n = 12 trials. (**b**) Cell viability of MCF-7 cells in drops accessed over 7 days without any media change. Up to 3 days, more than 90% of cells survived. From day 4, the viability of cells decreased. Cell viability analysis was based on 1287 cells, with each drop containing ~10 cells. (**c**) Changes in drop volume stored in microfluidic device over 7 days. The drop volume (V) compared to the initial drop volume (V_0_) is less than 11% over 7 days. The average volume ratios (V/V_0_) and standard deviations were calculated from 3 replicates.

A potential concern of the MCI technology for drug testing is that the drop environment might adversely affect the viability of tumor cell suspension. For example, MCF-7 cells that are cultured in the laboratory in the adherent state, may undergo anoikis in the microfluidic drop environment, and therefore potentially introduce bias in our drug testing data. To address this concern, we conducted microfluidic experiments with CCRF-CEM leukemia cells whose natural as well as laboratory culture environment is liquid media. This allowed us to compare the level of anoikis of these CEM cells with that of MCF-7 cells incubated in microfluidic droplets. As shown in Fig. 3a, both the MCF-7 and CEM cells showed greater than 90% viability in droplets across 24 hours. Statistical analysis reveals that the percentage viability between the two cells lines is indistinguishable, suggesting that the level of anoikis is not very different between MCF-7 and CEM.

We also conducted long-term viability studies for up to 7 days, with MCF-7 cells in the microfluidic drops (see Fig. 3b). We observed that more than 90% cells are viable up to 72 hrs. On days 4, 5, 6 and 7, we found that cell viability is 75, 62, 43 and 21% respectively. We also recorded changes in drop volume due to evaporation, and find that the change in volume in 72 hrs is less than 10%, after which the drop volume remains almost unchanged (Fig. 3c). Thus, significant reduction in cell viability after day 3 is potentially due to lack of nutrients and anoikis. These results suggest that cells can be cultured successfully for drug testing up to 72 hrs, using the MCI technology.

### Comparison of performance of MCI technology against multiwell plates

**Figure 4:**
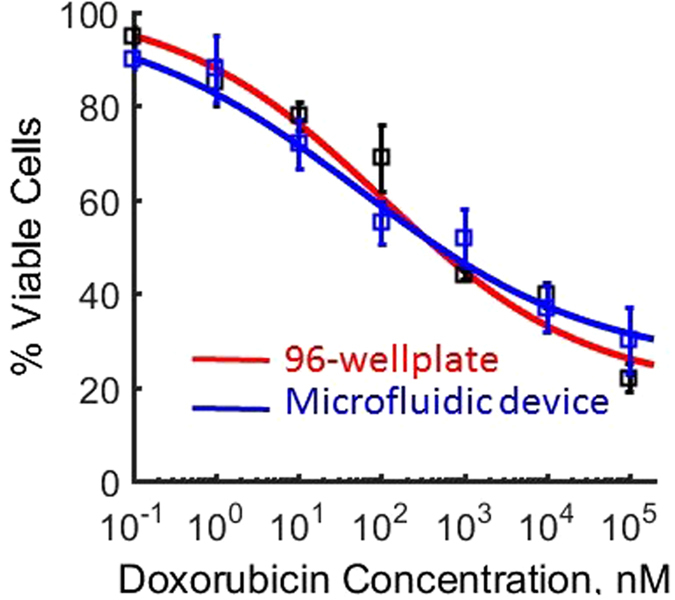
Comparison of performance of MCI technology versus standard multiwell plates. Dose response curves with doxorubicin and MCF-7 cells were generated using both approaches, after 24 hrs of incubation. Cell viability analysis at each drug concentration was based on ~600 cells, in both the approaches. Each data point represents the mean response and the error bar corresponds to standard deviation from 3 replicates. The solid line is the average dose response curve, which was generated by fitting the standard Hill equation. The half maximal inhibitory concentrations (*IC*_*50*_) for 96-wellplate and microfluidic device are not statistically significant (p > 0.05).

To assess the performance of our MCI technology vis-à-vis conventional method, we conducted a dose response assay using multiwell plates and the MCI technique, and compared the two methods. Muliwell plates are the most commonly used approach for conducting cell-based drug assays, Fig. 4 shows the comparison between the two approaches. We find that the dose-response curve obtained from the MCI technique is statistically indistinguishable from that of the 96-well plate data. This result suggests that the performance of our approach is comparable to that of multiwell plates, but with marked advantages of (i) isolating single cells without much cell loss and (ii) conducting dose-response assays with ~O(10^2^) cells, rather than the O(10^4^) cells that are typically used in multi-well plates.

### Kinetics of Dox uptake and its relationship to tumor cell apoptosis

Given that the MCI technology has the capacity to isolate single cells and the microfluidic drop environment does not adversely affect cell viability, we studied the kinetics of uptake of doxorubicin by individual breast cancer cells. Doxorubicin can induce cancer cell death through different mechanisms: (i) inhibition of topoisomerase II^47^; (ii) intercalation to base pairs of the DNA double helix, blocking the synthesis of DNA and RNA, and DNA strand scission^48^; (iii) binding to cellular membranes to alter fluidity and ion transport; and (iv) generation of semiquinone free radicals and oxygen free radicals through an enzyme-mediated reductive process^49^.

Despite the ability of Dox to induce cell death through several mechanisms, breast cancer cells (MCF-7) can still show drug resistance to Dox after first chemotherapy^50^,^51^. The uptake rate of a drug by cancer cells is usually controlled by the resistivity of the cell population^5^,^52^. As a result, there is a need to identify which cells in a population are resistant to Dox uptake and how the uptake kinetics correlates with cellular apoptosis. Such investigations are difficult to pursue with bulk drug assays. Microfluidic approaches are suitable for single cell drug assays^53^,^54^, however, a survey of literature shows that Dox uptake kinetics has not been correlated to apoptotic susceptibility in MCF-7 cells. Given that we have demonstrated effective isolation of single cells using MCI technology, here we study single-cell kinetics of Dox uptake, identify Dox sensitive and insensitive cells and establish a correlation between accumulation of Dox and apoptotic susceptibility.

**Figure 5:**
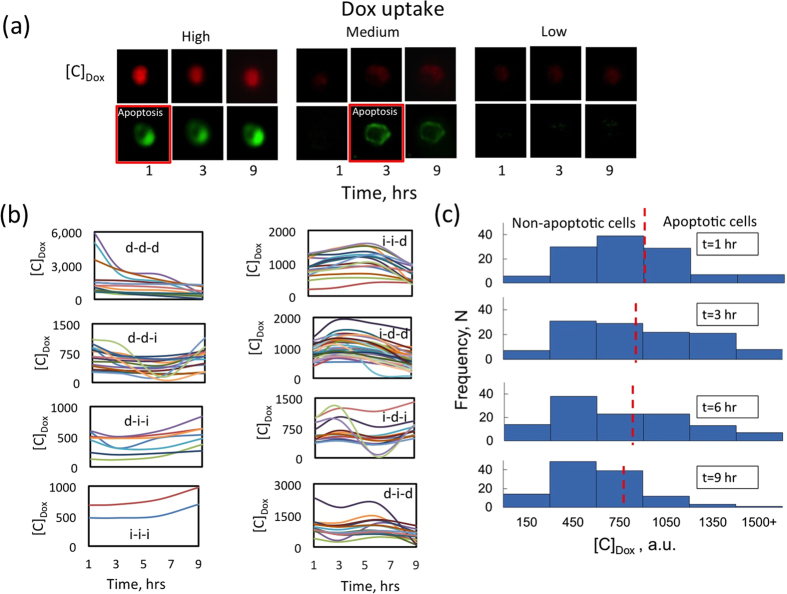
Uptake kinetics of doxorubicin in single breast cancer cells and correlation to apoptotic susceptibility. (**a**) The top row images show representative images of Dox fluorescence in the nucleus (red) and the bottom row shows the corresponding co-stained images for apoptosis (green) for the same cell inside a droplet. The red box highlights the timepoint at which apoptosis was noted. Based on the Dox concentration after 1 hour of incubation, all cells were categorized in three groups defined as: high ([C]_dox_ > 900 au), medium ([C]_dox_ = 301–900 au) and, low ([C]_dox_ < 300 au). (**b**) Dox uptake patterns of 118 single cells over 9 hrs. Total 8 different Dox uptake patterns were observed. Heterogeneity of individual MCF-7 cells was observed in terms of intracellular Dox uptake (represented by Dox concentration in arbitrary unit, au). The legends of subplots indicate the type of Dox uptake pattern; where‘d’ and ‘i’ represent decrease and increase in Dox concentration respectively. (**c**) Distribution of intercellular Dox concentration at four different time points. The red dashed line represents the critical Dox concentration needed for cellular apoptosis delineating the surviving and non-surviving cells.

To quantify Dox uptake, the amount of auto fluorescence due to presence of Dox in the nucleus of individual cells was measured from droplets that contained only single cells (n = 118) over a period of 9 hrs. Similar to previous studies^53^,^55^, we observed significant heterogeneity among MCF-7 cells in terms of Dox uptake and retention. Figure 5a shows representative images of Dox accumulation in the nuclei of individual tumor cells (in red) co-stained with Annexin V (in green) for apoptosis. In general, we observe that cells that uptake high amounts of Dox undergo apoptosis. Importantly, we found eight different Dox uptake and retention profiles depending on how the Dox concentration, [C]_Dox_, changes between different arbitrary time points of 1, 3, 6 and 9 hrs. As shown in Fig. 5b, we observed cells in which [C]_Dox_ continually decreases (d-d-d) or increases (i-i-i) or other combinations (*e.g.* d-d-i; d-i-i; i-d-d; i-i-d; i-d-i and, d-i-d). Among these most of the cells showed i-d-d (27%), d-d-i (15%) and, i-i-d (14%) uptake profiles and very few cells showed i-i-i (2%) and d-i-i (6%) profiles. Collectively, these results indicate significant heterogeneity in the uptake kinetics of Dox in MCF-7 cells.

Given the heterogeneous uptake profiles, we sought to investigate the correlation between Dox uptake and apoptotic susceptibility. We used two-color fluorescence imaging to establish this relationship and representative images are shown in Fig. 5a. We evaluated if there was a critical Dox uptake concentration at which tumor cells undergo apoptosis, irrespective of whether they uptake the drug slowly or rapidly. To address this, in Fig. 5c we plot the [C]_Dox_ distribution at different time points and identified the [C*]_Dox_ ≈ 825–900 at which apoptosis is initiated (vertical lines in Fig. 5c). Interestingly, we find that the critical intracellular Dox concentration needed to trigger apoptosis decreases only slightly with increase in incubation time. However, the percentage of apoptotic cells increases significantly from 35% to 58% at time points of 1 hr and 9 hr respectively. Taken together, these results suggest that a critical intracellular concentration of the chemotherapy drug Doxorubicin is needed to induce cell death in MCF-7 breast tumor cells and cells that do not uptake the critical Dox concentration survive.

The surviving and non-surviving cells show interesting uptake patterns. The non-surviving cells undergoing apoptosis uptake a Dox concentration higher than [C*]_Dox_ at initial time points, but the Dox concentration decreases towards the later time points. The surviving cells mostly follow the d-i-i, d-d-i, i-i-i, and i-d-i patterns, indicating that towards later time points the Dox concentration increases, but still remaining mostly below [C*]_Dox_.

### Isolation and drug response of clustered tumor cells (Cl-TC)

Most of the CTCs in patients are found as single cells. However, a very small fraction (2–5%) of CTCs are found as clusters with the number of cells within a cluster ranging from 2–50^19^,^30^. These clusters show 50 times more metastatic potential than single CTCs^19^,^56^,^57^,^58^. A major issue with handling CTC clusters is their sensitivity to rupture under shear. For example, the CTC-cluster chip uses an estimated shear rate of ≈700 s^−1^ to isolate tumor cell clusters from cancer patient blood^30^. The shear rate during the pipetting and loading/trapping process in the MCI technology is about 10–20 s^−1^, making it a promising candidate for handling clusters of tumor cells. We therefore generated clusters of MCF-7 tumor cells^30^ and tested the capability of the MCI method to efficiently encapsulate these clusters in the droplet array and conduct drug assays. We focused on addressing two questions: (i) What is the probability of generating clusters containing a given number of cells (ii) Do single tumor cells show different drug response than cells present in clusters?

### Size distribution of tumor cell clusters

**Figure 6:**
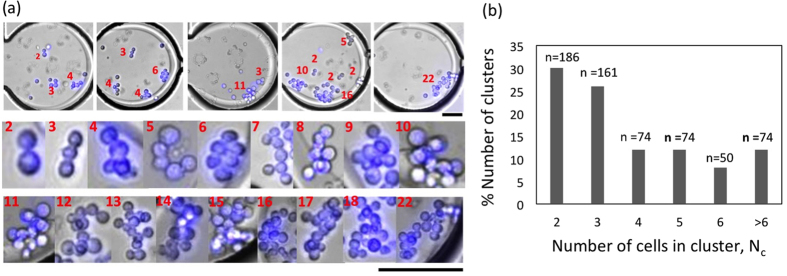
Morphology and distribution of tumor cell clusters of a given size. (**a**) Isolation of tumor cell clusters along with single tumor cells in trapped drops of volume 30 nl (top). Physical morphology of different sizes of cell clusters (bottom). The number of cells in the cluster varies from 2–22. Each image is composite image of bright field (grey) and fluorescence (DAPI-stained blue nuclei). Scale bars represent 100 μm. (**b**) Probability distribution of clusters of different sizes. Data was taken from a total of 360 drops.

To generate clusters, we chose a stock cell density such that the average number of cells per droplet, λ = 10. The detailed protocol for preparing clusters is discussed in the Experimental section. Using this protocol, we trapped both clustered tumor cells (Cl-TC) and single tumor cells (Sl-TC) in drops (Fig. 6a, top). As shown in the images of Fig. 6a, we obtain clusters with N_c_ = 2–22 cells, where N_c_ is the number of tumor cells in a cluster. To characterize the probability distribution of obtaining a cluster with a given N_c_, we interrogated 360 drops (from 6 microfluidic devices) and the data is shown in Fig. 6b. We find that clusters with N_c_ = 2 and 3 are the most predominant with probabilities of 30% and 26% respectively. Tumor cell clusters with N_c_ ≥ 4 have a lower probability of occurrence, about 1–12%. In addition, we note that the cluster size probability distribution depends on how cells detach during trypsinization and the extent of pipette-induced mixing, since the cluster distribution obtained from each of the 6 devices were not the same (data not shown).

### Drug response of single tumor cell (Sl-TC) vs. clustered tumor cell (Cl-TC)

**Figure 7:**
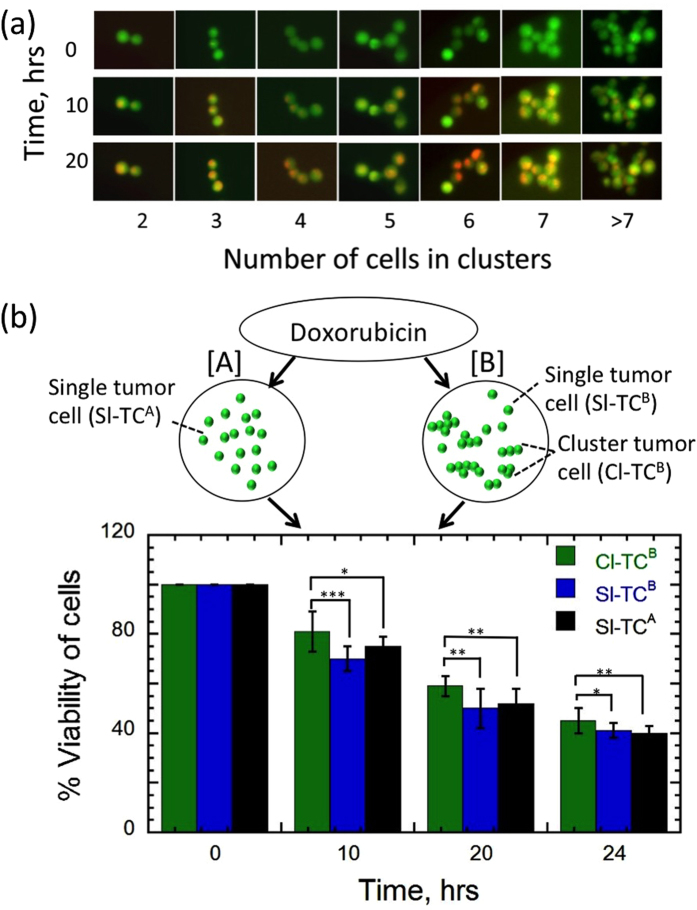
Comparing the viability of clustered tumor cells (Cl-TC) and single tumor cells (Sl-TC) to doxorubicin. (**a**) Images showing the viability of tumor cells in clusters of different sizes when exposed to 10 μM dox. Each image is a composite rendition showing live (green) and dead (red) cells. (**b**) The viability of two batches of MCF-7 cells were measured over time when exposed to 10 μM doxorubicin. Batch [A] contained only single tumor cells (Sl-TC^A^, n = 2275 and Batch [B] contained a mixed population of single tumor cells (Sl-TC^B^, n = 1054) and clustered tumor cells (Cl-TC^B^, n = 1621 cells out of 620 clusters). To test whether the clustered cells showed different response to drug compared to single cells, we performed standard unpaired t- test with 95% confidence interval at 10, 20 and 24 hrs. Statistical significance (p value) is represented with asterisks (*) with the following convention - *P < 0.05, **P < 0.01, ***P < 0.001. For all time points, cells in clusters (Cl-TC^B^) showed statistically higher viability than the viability of single individual cells [(Sl-TC^A^) and (Sl-TC^B^].

Figure 7a shows representative images of clusters of different sizes that have been exposed to Dox. In general, we find that a significant number of clusters contain live cells even after 20 hrs of drug exposure. To investigate whether isolated cells show a different drug response than cells present in clusters, we tested the response of Dox on two different batches as shown in Fig. 7b. Batch [A] contained only single tumor cells (Sl-TC^A^) and Batch [B] had a mixture of clustered tumor cells (Cl-TC^B^) and single tumor cells (Sl-TC^B^).

We assessed cell viability at 0, 10, 20 and 24 hrs in both batches. To test whether the clustered tumor cells show different drug response than single cells, we performed standard unpaired t- test for time points 10, 20 and 24 hrs. At these three time points, clustered tumor cells showed statistically higher viability than that of the independently generated single cell population as well as the co-existing single cell population. We checked for anoikis with clusters, and we did find it to contribute significantly to cell death (see Fig. S1). Thus, even though the single and the clustered cells were from the same population, the number of cells surviving drug exposure depended on whether they were present as individuals or clusters.

The Dox response of tumor cell clusters is different from that of single cells probably because the uptake kinetics of the drug could be different for a group of cells than individuals, especially when they are confined in microfluidic droplets. It is also possible that the overall drug resistivity of clustered tumor cells might be different compared to the resistivity of individual cells. From a cancer metastasis perspective, our result suggests that a CTC cluster has a better chance of surviving chemotherapy drug treatment than a single CTC.

## Conclusions

Patient-derived cells from human tissues and biofluids are increasingly being used to predict patient outcomes as they are a superior model of the *in vivo* situation^10^,^13^,^16^. For example, recent advances to separate CTCs from blood have opened up opportunities for downstream assays of rare CTCs. However, to harness the full benefits of these downstream assays of rare CTCs requires manipulating a small number of cells without their loss during handling. Even loss of one diseased CTC from a pool of cells, might incorrectly predict the patient outcome. In this context, our simple *MCI* method offers benefits of i) reproducible sample discretization, ii) control over distribution of minute amount of cells/clusters without much loss of cells, iii) assessing heterogeneity at single cell level, iv) easy identification of drug response and, v) long time observation of intracellular events while preserving identity of each cell.

To demonstrate the power of the method, we have conducted proof-of-principle drug studies and provided useful insights into the response of single isolated tumor cells and clusters of tumor cells. Importantly, we have shown that individual cancer cells show a rich variety of drug uptake patterns and that clusters are more resistant to drug treatment. These results obtained from *in vitro* tumor cell lines need to be validated with actual CTCs from patient samples. The capabilities of the MCI technology can be further expanded to replace media from the droplets^44^ for long term drug studies and also characterize the influence of drugs on co-culture of cells (e.g. different proportions of tumor and stromal fibroblast cells^59^.

## Methods

### Fabrication of microfluidic devices

The design of the devices used in this study is identical to that used in our previous works^43^,^44^. Briefly, the device contains a microfluidic network of 60 loops (Fig. 1). Each loop has a bypass channel and a lower branch containing a trap that can park a droplet of volume 30 nL (Fig. 1a). In this microfluidic device^44^, all the channel widths and diameters of the trap chambers are 200 μm and 450 μm respectively. The height of all the features in the device is 170 μm. Microfluidic devices were fabricated using soft lithography^60^. Polydimethyl siloxane (PDMS) prepolymer and curing agent were mixed in a 10:1 ratio, degassed and poured on the mold and cured for a minimum of two hours at 65 °C. Subsequently the PDMS replica was cut with a scalpel and peeled. Inlet and outlet reservoirs were defined by punching holes. The replica was placed on a PDMS coated glass slide that was partially cured in the oven at 65 °C for 10 minutes. The entire assembly was further cured at 65 °C for two hours to create an irreversible seal.

### Cell culture

The human breast cancer cell line (MCF-7) obtained from American Tissue and Culture Collection (ATCC, Manassas, VA, USA) was maintained and grown in DMEM medium containing 10% fetal bovine serum, 1% Sodium Pyruvate, and 1% Penicillin/Streptomycin at 37 °C in 5% CO_2_. Cell culture chemicals were purchased from Fisher Scientific. Different cell densities of MCF-7 cells were used as samples in the cell assay experiments. For all experiments, the initial cell concentration was 1 × 10^6^ cells/ml and desired final concentrations were achieved by diluting with DMEM media.

### Preparation of cell samples

To produce the cell samples for single tumor cell assays, we used a cell concentration of 0.35 × 10^6^ cells/ml. The dilution and staining process were done by pipette mixing multiple times (at least 10 times) followed by vortex mixing to ensure that no clustered cells are disrupted.

To produce clustered tumor cell clusters (Cl-TC), we used the same concentration as the single cell assays, but we modified the mixing process to preserve cell clusters/groups. The total dilution and staining process were done by only three times mixing with pipette. This ensured mixing without significantly disrupting cell clusters. However, in this process each of the traps of the microfluidic device contained a mixture of clustered tumor cells and single tumor cells.

### Drug assays

We conducted drug assays on the tumor cells in our microfluidic device, using the anti-cancer drug doxorubicin hydrochloride (Dox) (Sigma Aldrich). For all assays, Dox was used at a concentration of 10 μM. Initially, Dox was mixed with distilled water to make a 1 mM stock solution of drug and stored at 4 °C. For all experiments, stock solution of Dox was freshly diluted with DMEM media to a concentration of 10 μM. For drug assays, Dox was mixed with the cell samples and immediately loaded into the microfluidic device and imaged. The drug mixing and loading process were done within 2 minutes. In between different imaging time points the microfluidic devices were incubated at 37 °C in 5% CO_2_.

### Pipetting procedure for loading cell samples

All the samples including cell suspensions and drug treated cells were handled in the microfluidic device using a multi-channel VIAFLO Electronic Pipette (0.5–12.5 μL, INTEGRA Biosciences Corp., NH, USA) and Grip-tips pipette tips (12.5 μL, INTEGRA Biosciences Corp., NH, USA). We tested several pipette tips (10 μL, 12.5 μL, 100 μL, 200 μL) and dispensed volumes (5 μL–10 μL) on a manual pipettor. We found that 12.5 μL, pipette tips with dispensed volumes of 7 μL worked the best in terms of uniform trapped volumes. The microfluidic devices were primed with a carrier fluid (FC-40, Sigma Aldrich) containing 0.001% w/w biocompatible fluorosurfactant (KrytoxFSH-PEG600-KrytoxFSH, RAN Biotechnologies, Inc., MA). Then, an off-chip oil-sample cartridge was prepared in the pipette tip using the multichannel automated pipette. The cartridge contains 5 μL of oil and 2 μL of sample. This oil-sample cartridge was then injected, using the electronic pipette, into the device that was prefilled with carrier fluid.

### Viability and apoptosis detection

LIVE/DEAD® Cell Imaging Kit (Life technologies) was used to quantify cell viability. The ‘Live’ and ‘Dead’ parts of the stain were mixed following the manufacturer protocol. The mixed solution was diluted 3 times with cell media. This 3x-diluted solution was mixed with cell samples at 50/50 volume ratio to stain the cells. NucBlue® Live ReadyProbes® Reagent (Life technologies) were used to identify cell nuclei to accurately quantify cell counts.

Apoptosis was detected using Alexa Fuor^®^ 488 Annexin V (Thermo Fisher Scientific). The dilution of cell samples and Dox were done with 1X Annexin- binding buffer (Thermo Fisher Scientific). Dox was mixed with the cell samples as described for other drug assays at a concentration of 10 μM. 25 μl of Alexa Fuor^®^ 488 Annexin V (Thermo Fisher Scientific) was added to 100 μl cell-drug samples as an early-stage apoptosis marker. The cell sample with the Alexa Fuor^®^ 488 Annexin V was incubated at room temperature for 15 min, loaded to the microfluidic device and then incubated at 37 °C in 5% CO_2_ for 45 mins. Fluorescence images (FITC and TRITC) were taken at time points of 1, 3, 6 and 9 hrs. In between imaging, the devices were incubated at 37 °C in 5% CO_2_.

### Image acquisition and processing

Brightfield and fluorescence images of cells in droplets were captured using Olympus IX 81 microscope (Massachusetts, USA) equipped with a Thorlab automated stage (New Jersey, USA) controlled by SlideBook 6.1 (3i Intelligent Imaging Innovations, Inc., Denver, USA). Images were acquired with a Hamamatsu digital camera (ImagEM X2 EM-CCD, New Jersey, USA), 10–60x objectives and standard FITC/DAPI/TRITC filters. The exposure times used in the experiments ranged from 20–430 ms.

The uptake of doxorubicin *i. e.* concentration of Dox, [C]_Dox_, was quantified from the time-lapse images of each cell. Image processing was done with ImageJ (http://rsb.info.nih.gov/ij/). The [C]_dox_ in arbitrary units (a.u.) was calculated after background correction by the following equation, [C]_dox_ = Mean fluorescence of cell − Mean fluorescence of background. A composite image from FITC (live) and TRITC (dead) filters was used to assess live/dead cells in ImageJ.

## Additional Information

**How to cite this article**: Bithi, S. S. and Vanapalli, S. A. Microfluidic cell isolation technology for drug testing of single tumor cells and their clusters. *Sci. Rep.* **7**, 41707; doi: 10.1038/srep41707 (2017).

**Publisher's note:** Springer Nature remains neutral with regard to jurisdictional claims in published maps and institutional affiliations.

## Supplementary Material

Supplementary Information
