# Supplementary material for: Microfluidic cell isolation technology for drug testing of single tumor cells and their clusters
Source: Sci Rep. 2017 Feb 2;7:41707. doi: 10.1038/srep41707 (PMC5288702; doi:10.1038/srep41707)
Supplement: Supplementary Information [file srep41707-s1.doc]

**Microfluidic cell isolation technology for drug testing of single tumor cells and their clusters**

Swastika S. Bithi and Siva A. Vanapalli*

Department of Chemical Engineering, Texas Tech University, Lubbock, TX 79409

**Supplementary Information**

***Viability of clustered tumor cells in drop environment.***

The utility of cell-based assays in microfluidic droplets with either Sl-TC or Cl-TC depends on the biocompatibility of the drop environment where cells are encapsulated in aqueous media surrounded by an oil phase. Therefore, we tested two batches ([A] and [B] in Fig S1) to determine the impact of the droplet environment on cell viability. Batch [A] contained single tumor cells (Sl-TC^A^) and batch [B] contained both single (Sl-TC^B^) and clustered tumor cells (Cl-TC^B^). Cell samples were prepared as described in the Methods section. Though MCF-7 cells are adherent cells, previous studies have shown that they remain functional in suspension culture for at least 24hrs^1^. Therefore, all of our assays were performed within 24 hrs after trypsin-mediated detachment. The cells were stained with live-dead marker and were loaded to 12 microfluidic devices containing 720 drops. We identified the viable cells at different time intervals over 24hrs using epifluorescence microscopy. We put the devices in water-filled omni-plates with lids and stored plates in the incubator between imaging. All drops were stable and spaced apart in individual traps, there was no need of delay lines or off-chip storage like other studies for long time storage ^2^. We found that less than 5% of the reagent in drops was lost for a 24 hr incubation period. For both batches ([A] and [B]) we retained more than 90% cell viability over 24 hours without media replacement (Fig. S1), confirming minimal adverse effects of our method on cell viability of both tumor cell clusters and individual cells.


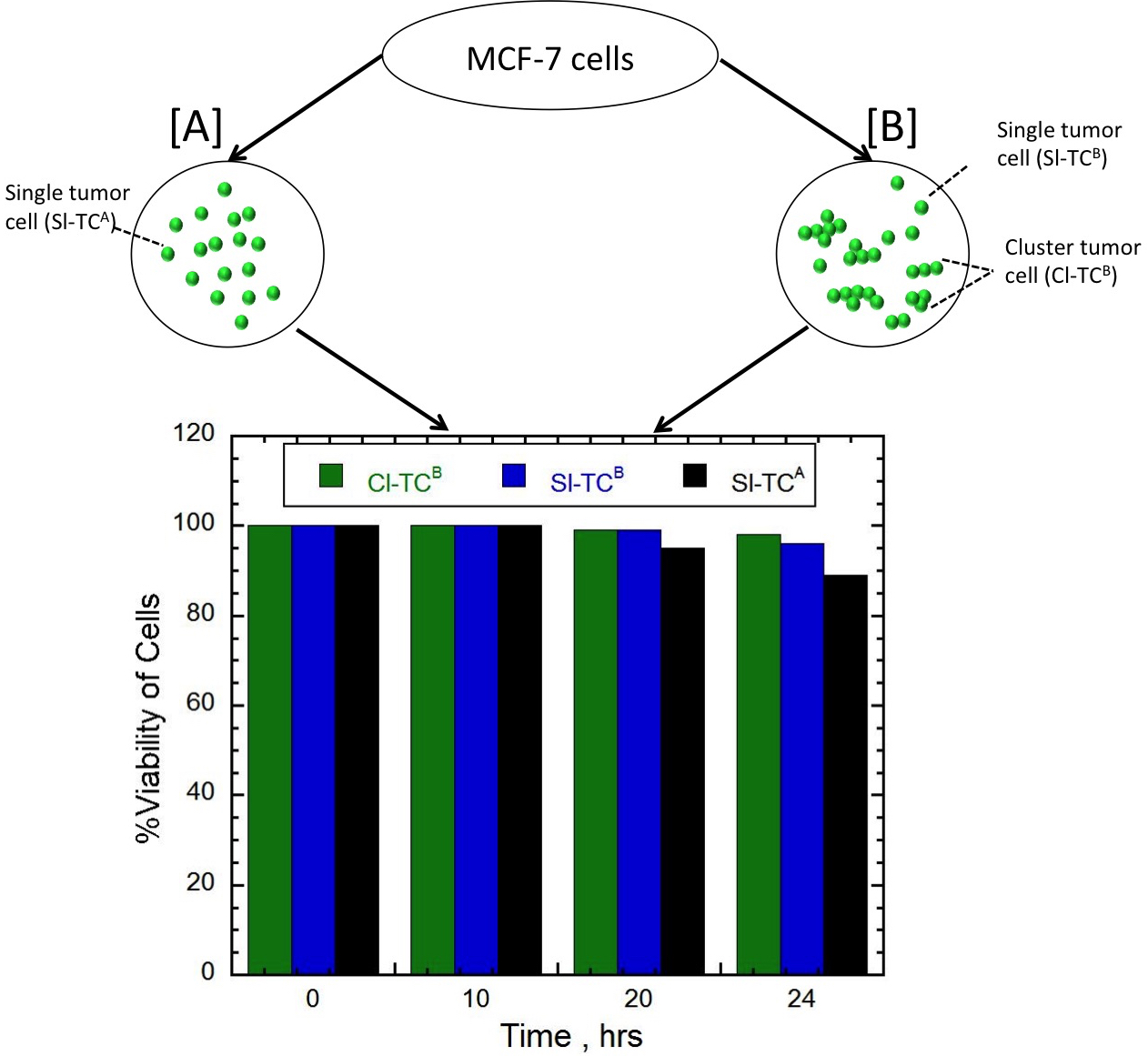


Figure S1. ***Viability of single and clustered tumor cells in drop environment*.** The viability of two batches of MCF-7 cells in 12 microfluidic devices was measured over 24 hrs. One of the batch [A] contained only single tumor cells (Sl-TC^A^) and the other batch [B] contained a mixed population of single tumor cells (Sl-TC^B^) and clustered tumor cells (Cl-TC^B^). More than 90% of cells from both batches survived after 24 hrs of incubation.

**References**

1. Pourreau-Schneider, N. *et al.* Estrogen response of MCF-7 cells grown on diverse substrates and in suspension culture: promotion of morphological heterogeneity, modulation of progestin receptor induction; cell-substrate interactions on collagen gels. *J Steroid Biochem* **21**, 763-771 (1984).

2. Brouzes, E. *et al.* Droplet microfluidic technology for single-cell high-throughput screening. *Proceedings of the National Academy of Sciences* **106**, 14195-14200 (2009).
